# Complete genome sequence of *Levilactobacillus brevis* 47f: a potential pharmabiotic strain

**DOI:** 10.1128/mra.00123-25

**Published:** 2025-12-10

**Authors:** Tatiana Koshenko, Elena Poluektova, Roman Yunes, Maria Marsova, Valery Danilenko

**Affiliations:** 1 Vavilov Institute of General Genetics, Russian Academy of Sciences 145227 , Moscow, Russia; Fluxus Inc., Sunnyvale, California, USA

**Keywords:** *Levilactobacillus brevis *47f, GABA, pharmabiotic

## Abstract

*Levilactobacillus brevis* 47f is a human-isolated strain known to possess antioxidant and antimucositic activities. We present the complete genome sequence of the strain *L. brevis* 47f, which consists of a circular chromosome (2,400,704 bp) and five plasmids.

## ANNOUNCEMENT

Lactobacilli are permanent inhabitants of the human intestinal microbiota with significant therapeutic potential. *Levilactobacillus brevis* 47f modulates gut microbiota (1), reduces oxidative stress (2), and protects against experimental mucositis (3, 4). *L. brevis* 47f was obtained from the Russian State Collection of Normal Microflora. Originally isolated in 2010 from the feces of a healthy woman in Central Russia, written informed consent was obtained, and no further ethics approval was required. A draft genome was previously sequenced (GenBank GCA_001010995.1); here, we report the complete genome.

The strain was cultured in 2 mL of de Man, Rogosa, Sharpe (MRS) broth (HiMedia, India) at 37°C for 18 h to stationary phase without shaking. Genomic DNA was extracted using the Wizard DNA Extraction Kit (Promega, USA) and purified with solid-phase reversible immobilization (SPRI) beads. DNA concentration and quality were assessed using a Qubit 4 fluorometer and Nanodrop ND-1000 (Thermo Fisher Scientific).

Whole-genome sequencing was performed using both Oxford Nanopore Technologies (PromethION) and Illumina (NextSeq 1000) platforms. A total of 452,296 Nanopore reads (1.3 Gb) and 5,000,000 Illumina paired-end reads (957.8 Mb) were obtained. The genome was sequenced with an estimated coverage of ~200× using combined Illumina and Oxford Nanopore reads.

For Illumina sequencing, 100 ng of extracted DNA was used to prepare libraries with the KAPA HyperPlus Kit (Roche, Switzerland) according to the manufacturer’s instructions. Libraries were purified with KAPA HyperPure Beads, and library size distribution and quality were evaluated using a high-sensitivity DNA chip (Agilent Technologies). Libraries were quantified using the Quant-iT DNA Assay Kit, High Sensitivity (Thermo Fisher Scientific). Paired-end reads of 100 bp were generated on a NextSeq 1000 platform using NextSeq 1000/2000 P2 Reagents (200 cycles) with a 2% PhiX spike-in control. Reads were quality-filtered using standard Illumina procedures, and error correction was applied during genome assembly using Pilon.

For long-read sequencing, DNA was not sheared or size-selected. Libraries were prepared with the Oxford Nanopore Technologies ligation sequencing kit, SQK-LSK109, and native barcoding expansion kit, EXP-NBD114, and sequencing was performed on a FLO-PRO002 flow cell using the PromethION platform. Reads were basecalled using Guppy v6.5.7. *De novo* assembly was performed using Flye v2.9.2 with default parameters, and the assembly was corrected with Illumina reads via Pilon. Circularity and replicon boundaries were determined from *de novo* assembly graphs generated by Flye v2.9.2. Completeness (98.52%) was assessed using CheckM v1.2.3. No rotation to a specific base was applied.

Annotation with the National Center for Biotechnology Information PGAP v6.10 (RefSeq)/v6.6 (GenBank) identified 2,694 genes, including 2,546 protein-coding sequences (CDS), 15 rRNA genes (5S, 16S, and 23S), and 71 tRNA genes. The final assembly (GCF_001010995.2, ASM101099v2) consists of a single circular chromosome and five plasmids (Table 1).

**TABLE 1:** Genome features and sequencing data of *Levilactobacillus brevis* 47f

Replicon	Size (bp)	GC content (%)	RefSeq	GenBank
Chromosome	2,400,704	46.0	NZ_CP145109.1	CP145109.1
Plasmid p47-1	20,350	39.5	NZ_CP145107.1	CP145107.1
Plasmid p47-2	23,228	42.0	NZ_CP145108.1	CP145108.1
Plasmid p47-3	61,382	42.5	NZ_CP145110.1	CP145110.1
Plasmid p47-4	64,655	41.0	NZ_CP145111.1	CP145111.1
Plasmid p47-5	63,507	42.5	NZ_CP145112.1	CP145112.1

Functional annotation identified genes involved in vitamin and amino acid biosynthesis, GABA production, antioxidant defense, and bile salt hydrolysis.

## Data Availability

The complete genome sequence of *Levilactobacillus brevis* 47f has been deposited in GenBank under accession number GCA_001010995.2. The raw sequencing reads are available in the NCBI Sequence Read Archive (SRA) under accession numbers SRR35983847 (Illumina NextSeq 1000) and SRR34797683 (Oxford Nanopore PromethION) associated with BioProject PRJNA280953 and BioSample SAMN03470252.
